# Dose-response and time-lagged effect of daily training load on athlete well-being during an international rugby series

**DOI:** 10.5114/biolsport.2025.139080

**Published:** 2024-05-07

**Authors:** Blair T. Crewther, Benjamin Serpell, Neill Potts, Liam P. Kilduff, Christian J. Cook

**Affiliations:** 1 School of Science and Technology, University of New England, Armidale, Australia; 2 Institute of Sport – National Research Institute, Warsaw, Poland; 3 School of Electrical Engineering and Robotics, Queensland University of Technology, Brisbane, Australia; 4 Hamlyn Centre, Imperial College, London, UK; 5 Geelong Cats Football Club, Geelong, Victoria, Australia; 6 Western Australian Institute of Sport, Perth, Australia; 7 A-STEM, School of Engineering, Swansea University, Swansea, UK; 8 Welsh Institute of Performance Science (WIPS), Swansea University, Swansea, UK

**Keywords:** Contact Sport, Trainability, Recovery, Adaptation, Stress

## Abstract

Rugby training and competition both impose a stress or training load (TL) affecting athlete well-being. Current understanding of the TL dose-response and time-lagged changes (i.e., delayed effects) in well-being is limited. We addressed these gaps using data from a 3-week international series. Twenty-two elite male rugby players were assessed 4–5 days a week for daily TL and well-being (i.e., mood, stress, soreness, fatigue, sleep quality). A distributed lag non-linear model was used to estimate the TL effect, at the within-person mean (358 A.U), +1SD (576 A.U) and +2SD (794 A.U), on well-being at 0–5 lag days. Average daily TL declined by -38% and -45% in weeks two and three (vs. week 1), respectively, with weekly fluctuations of +27% to -58% (vs. Monday training). The well-being subscales oscillated around a stable baseline. Compared to mean-centered scores, a significant decline in mood and sleep quality (-0.6 to -2.0 units; -9 to -30%) emerged at all TLs, with a delayed shift at higher loads. Elevated stress, soreness, and fatigue responses (up to 3.7 units; 76%) emerged with increasing daily TLs, including a biphasic rise in stress and fatigue at higher loads. In conclusion, we gained detailed insight into physical stress and the temporal sequence of well-being during an international rugby series. Different daily TLs predicted adverse well-being responses (i.e., declining mood and sleep quality, rise in stress, soreness, fatigue) that varied in lag timing, duration, and magnitude. Explicating these associations can assist weekly planning and strategies to optimize recovery, performance, and team success.

## INTRODUCTION

Rugby union, henceforth referred to as rugby, is a collision sport characterized by intermittent, high-intensity activities (e.g., sprints, collisions) interspersed with periods of low-intensity activities and rest [1, 2, 3]. Training for rugby imposes a stress or training load (TL) affecting well-being in a dose-dependent way [2, 4, 5]. For example, a high TL reportedly promotes a deterioration in next-day sleep quality, motivation, fatigue, stress, and appetite versus a low TL [4]. Furthermore, a typical training week in professional rugby players, which can include multiple daily sessions, revealed significant fatigue and soreness 1–2 days after baseline (day 1) testing, with small to moderate effect-size shifts in fatigue, soreness, and sleep quality 1–6 days later [3]. Despite these findings, temporal knowledge of athlete recovery is often limited by next-day comparisons [2, 4, 5]. Consequently, at the elite playing level, a more detailed longitudinal examination of TL and well-being changes over time is needed.

Rugby matches, which account for ~5–11% of all rugby-related activities [1], present another strong adaptive stimulus with timelagged outcomes (i.e., delayed effects over several days after a given stimulus). This includes post-match changes in mood, sleep, muscle damage, and fatigue metrics, alongside performance and hormonal state [6, 7, 8, 9]. Reviews of rugby literature indicate that psychological well-being (e.g., stress, fatigue) is highly responsive to competition and it’s outcome, but recovers within 2–3 days [10, 11]. In contrast, post-match perceptions of physical recovery (e.g., soreness) might take four or more days before restoration to baseline values [12]. This difference in psychological and physical recovery is likely due to exercise- and impact-related muscle damage [11]. Surprisingly, few studies have quantified individual perceptions of competitive stress across repeated matches [7]. This, we believe, is a prerequisite to discern the time-course of psychological and physical recovery, relative to diverse match loads or dose-response equivalency under ecological conditions.

Tournament play presents another unique stressor characterized by training and match loads of varying intensity, duration, and complexity, along with planned and weekly TL variation [1, 13, 14, 15]. These demands are frequently coupled with short recovery periods between training days and competition; dramatically affecting athlete well-being and recovery. Rugby studies have linked different TL indicators to well-being, stress, and recovery in such settings [14, 16, 17], but findings are generally restricted by the reporting of pooled (e.g., seasonal or weekly) effects. To date, no research has sought to illuminate the TL and well-being associations (i.e., dose-dependencies, time-lagged interplay) at the daily level during elite rugby tournament play. Since elite athlete monitoring and exercise prescription often resides in a narrow assessment-feedback window, understanding these intricacies could provide a stronger basis to guide decisions to optimize recovery, performance, and team success.

To address gaps in the literature, we investigated the dose-response and time-lagged effects of daily TL on athlete well-being (i.e., mood, stress, soreness, fatigue, sleep quality) during a 3-week international rugby series. Our primary goals were to: (1) profile daily TL and wellness fluctuations between and within study weeks, and (2) model the daily TL and well-being associations at varying dosages and time lags. To capture significant or meaningful effects in an elite rugby context [3, 12, 18], we tested the impact of three daily TLs (i.e., within-person mean, +1SD and +2SD above the within-person mean) on well-being across 0–5 lag days. No firm hypotheses were made in relation to these goals, as the rugby series profiling and lagged analyses were exploratory in nature and contingent upon data collected herein.

## MATERIALS AND METHODS

### Participants

Twenty-two elite male rugby players, who formed part of a national (Scotland) training squad preparing for an international series in 2010, were assessed in this research. To ensure robust model estimates, we set a minimum number of TL (8 or more) and well-being (12 or more) observations for study inclusion, which also excluded injured players in our final analyses. Our sample comprised of 10 backs and 12 forwards with a mean (± SD) age, height, and body mass of 27.6 ± 3.4 years, 1.88 ± 0.09 m, and 102.2 ± 14.1 kg, respectively. The participants entered a training-camp environment upon selection, and this ensured some control in terms of physical training and other environmental factors (e.g., nutritional intake, meal timing, sleep, travel). Each participant received a full health and medical screening before study commencement. Written informed consent was given prior to data collection, but after a full briefing of the study aims, procedures, and potential benefits. Ethical approval (Number 2010.001R) was granted from the Swansea University Human Research Ethics Committee, Swansea.

### Study design

A longitudinal, single-group, observational design was employed to achieve the study goals. The participants were monitored across a 3-week international rugby series played in the northern hemisphere autumn test window, involving three matches against southern hemisphere teams. Daily TL was assessed 4–5 days per week across all training and match activities. Psychological (i.e., mood, stress) and physical (i.e., soreness, fatigue, sleep quality) well-being were assessed at a similar weekly frequency. In the first instance, each variable was described in terms of changes within, and differences between, study weeks. Next, we applied a distributed lag non-linear model (DLNM) to estimate the bi-dimensional lag-response associations [19] on the pooled dataset. Specifically, we predicted the well-being responses at 0–5 lag days following three incremental daily TLs.

### Methodology

Training days were scheduled for the Monday, Tuesday, Wednesday, and Friday of each week (i.e., 1–3 sessions a day, 30–90 mins a session), with Thursday and Sunday allocated as rest days. Some training adjustments were made depending on the match outcome and team performance. For example, an extra rest day was prescribed on Monday in the last week. Training load was monitored across all planned sessions (i.e., team and skills-based workouts, conditioning sessions, exercise stress-testing, gym workouts) using the session rating of perceived exertion (sRPE) method, anchored on a 0–10 Likert scale [20]. This metric is widely used in rugby research and practice to track individual perception of physical and physiological stressors [1]. Briefly, the participants provided a sRPE within 30 mins of each training session, which was multiplied by activity duration (in minutes) to determine TL in arbitrary units (A.U) [20]. Where two or more sessions were completed per day, the TLs were summed to derive a single daily TL representing physical stress on that day.

The three international matches were played on consecutive Saturdays at the home venue, or a nearby venue, for this team. For those participants selected to play, a daily TL was computed by multiplying the sRPE by actual time played (in minutes) [13]. Playing time was recorded by coaching staff on game day and later verified using an online resource (http://en.espn.co.uk/rugby/). Because of restricted post-match access to the studied athletes, the sRPE data were collected the next day prior to team breakfast. The total number of TL observations, including all training and match days, was 281 (participant range = 8–14).

Athlete well-being was self-assessed before breakfast (served around 8–9 am) on the Tuesday, Wednesday, Friday, Saturday, and/or Sunday of each week. Wake time was not strictly assessed, but the athletes arose before breakfast. Likewise, bed time was not prescribed, but rather self-selected to ensure sufficient sleep was obtained for training and matches. Participants completed a simple inventory rating their current psychological (i.e., mood, general stress) and physical (i.e., muscle soreness, general fatigue, sleep quality) state [2, 4, 5, 21]. Each subscale was scored on a 10-point Likert scale, anchored from one (extremely low / poor) up to 10 (maximal / excellent). Single-item perceived measures are widely used in rug-by [1, 4, 13], and often exhibit greater sensitivity than objective markers to detect small individual changes in recovery and fatigue [4]. The total number of mood, stress, soreness, fatigue, and sleep quality observations was 323–324 (participant range = 12–16).

### Statistical analyses

Study data were analyzed using R software [22]. First, we described the daily TL and well-being trajectories, after plotting each time series over the 21-day period. To better represent well-being dynamics, each time series was smoothed using a generalized additive model [23]. Second, descriptive statistics were calculated for each variable, including within-person means and SDs, along with an intraclass correlation coefficient (ICC) to assess measurement reliability. The ICCs were interpreted as being poor (< 0.50), moderate (0.50 to 0.75), good (0.75 to 0.90), and excellent (> 0.90) [24]. Finally, within-person Pearson correlations were computed to assess bivariate relationships between study variables, that we defined as a weak (0.20 to < 0.40), moderate (0.40 to < 0.60), strong (0.60 to < 0.80) or very strong (0.80+) effect size [25].

To examine the dose-response and time-lagged effect of daily TL on athlete well-being, we ran a series of DLNMs in the dlnm package [19]. In a two-step process, we first constructed a cross-basis for each comparison; a bi-dimensional space of functions describing the association along the spaces of predictor and lags [19]. The cross-basis was fitted with a natural cubic spline for lag-response, quadratic spline for exposure-response, and a maximum lag of five days. Participant was added as a group factor. One requirement (for a predictor) is an equally-spaced, complete, and ordered time series [19]. To achieve this, missing daily TLs were allocated a value of 1 and actual daily TLs corrected by the same amount. Once constructed, the cross-basis function was entered into a random intercept, linear mixed-effects model to predict the daily TL and well-being associations at each nominated lag length.

The DLNM results are plotted as lag-response curves over 0–5 days (at 0.5 day or 12-hourly intervals). Estimates were derived for three daily TLs (358, 576, 794 A.U) determined from the within-person descriptive results: see Table 1. We chose a reference point of 140 A.U (-1SD below the within-person mean), as each relationship was modelled with a non-linear function with no obvious reference value. The well-being estimates at each lag were mean-centered before plotting, as the default software procedure. As such, y-axis values above and below zero respectively indicate higher and lower well-being scores from study-averaged values. All predictions are presented with a 95% confidence interval (CI). A 95% CI band that excludes zero can be interpreted as representing a significant window.

**TABLE 1:** Within-person descriptives for the daily training load and well-being measures.

Measure	N	Mean	SD	ICC
Daily training load (A.U)	281	358	218	0.02
Mood (1–10)	324	6.77	1.17	0.24
Stress (1–10)	323	4.51	1.04	0.31
Soreness (1–10)	324	4.96	1.34	0.23
Fatigue (1–10)	324	4.90	1.32	0.19
Sleep quality (1–10)	324	6.09	1.55	0.25

N = observations, ICC = intra-class correlation coefficient.

## RESULTS

**FIG. 1:**
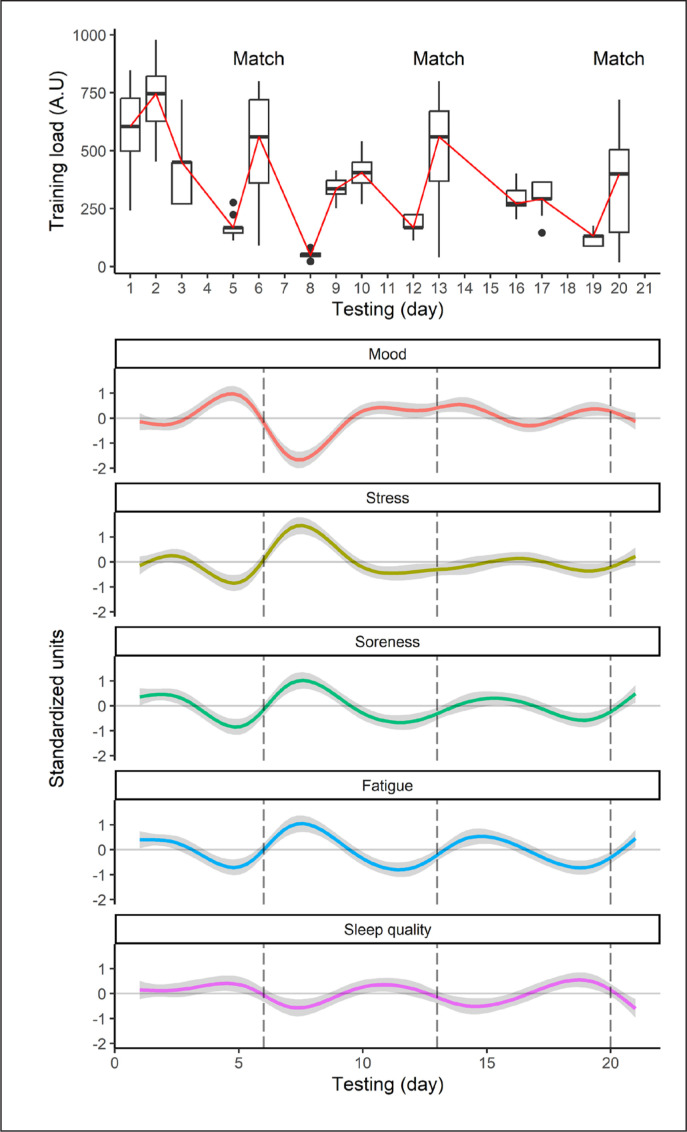
Time-series plots for daily training load and each well-being measure across the 3-week international rugby union series. Daily training load is presented (see top figure) as median values with an interquartile range. The well-being measures are depicted (in bottom figures) in standardized units, with solid lines representing the smoothed trajectory and shaded area the 95% CI. The dashed vertical lines indicate each match played.

The time-series plots are illustrated in Figure 1. A higher average daily TL emerged in week one (489 ± 244 A.U) than in weeks two (301 ± 186 A.U) and three (267 ± 155 A.U), due to a decline in training intensity and/or frequency; see methods above. Daily (match) TLs were similar in weeks one (519 ± 232 A.U) and two (510 ± 223 A.U), but lower in week three (364 ± 231 A.U). These outcomes followed a substantial defeat (by 46 points) before two close victories (by 4 and 3 points). On average, daily TL tended to rise and fall within a training week (Monday = 367 ± 296 A.U, Tuesday = 465 ± 228 A.U, Wednesday = 376 ± 107 A.U, Friday = 154 ± 40.5 A.U), before increasing on match day (Saturday = 459 ± 237 A.U). Mood and sleep quality tended to rise (up to 1SD) a day before each match, before falling below (down to -1.7SD) the study average or baseline scores 1–2 days after, and returning to baseline 3–4 days before the next game. Perceptions of stress, soreness, and fatigue showed a reversal in these patterns, with lower scores (down to -0.9SD) a day prior to competition, rising values (up to 1.5SD) 1–2 days after, and a mid-week return to baseline.

Table 1 summarizes the descriptive and reliability statistics for each study variable. The ICC for daily TL was trivial (0.02), meaning that trait reliability for this outcome was extremely poor. Interpreted another way, 98% of the variance in daily TL is explainable by state factors; a result coinciding with the considerable TL range (18–979 A.U). The well-being ICCs were stronger (0.19 to 0.31), but still poor overall, and again indicate more variance (69–81%) at the state level. In summary, we found that the predominant source of measurement variation in the current context was day-to-day (with-in-person) shifts, whilst trait-like (between-person) differences was a relatively minor source.

Within-person changes in daily TL were not significantly related to any well-being measure (see Table 2). We did find significant (*p* < 0.001) interrelationships between all well-being subscales, varying between weak and strong effects. Within-person perceptions of sleep quality and mood state tended to rise and fall together (i.e., positive relationships), as did stress, soreness, and fatigue. A rise in sleep quality and mood was accompanied by a decline in stress, soreness, and fatigue (i.e., negative relationships). These linkages are consistent with the plotted time series, whereby covarying patterns of change between two variables yielded positive relationships and opposing patterns produced negative relationships.

**TABLE 2:** Within-person correlations between the daily training load and well-being measures.

Measure	Sleep quality	Fatigue	Soreness	Stress	Mood
Daily training load	0.04	0.15	0.08	0.04	-0.03
Mood	0.37^*^	-0.48^*^	-0.49^*^	-0.53^*^	
Stress	-0.27^*^	0.54^*^	0.43^*^		
Soreness	-0.24^*^	0.67^*^			
Fatigue	-0.24^*^				

Within-person correlations are significant at

* *p* < 0.001

**FIG. 2:**
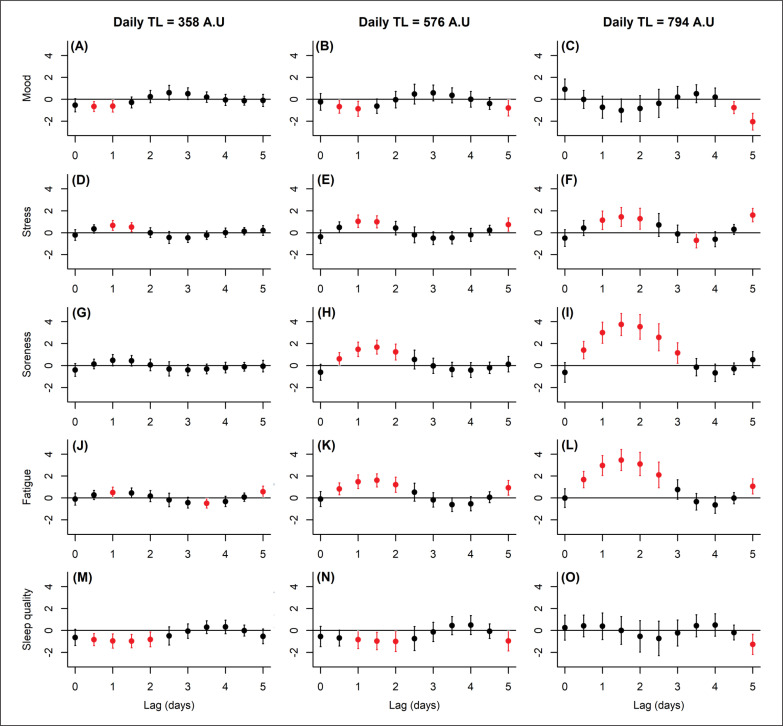
Effect of daily training load at three dosages on each well-being measure over lag periods of 0–5 days. The well-being subscales were centered before analysis, so that the y-axis is interpretable as deviation from study-averaged values. Error bars indicate the 95% CI with significant (95% CI band excluded zero) effects highlighted in red.

The lag-response associations are displayed in Figure 2. A significant decline in mood (-0.6 to -2.0 units; -9 to -30%) was seen at a daily TL of 358 A.U (0.5–1.0 days, 2A), 576 A.U (0.5– 1.0 days, 2B), and 794 A.U (4.5–5.0 days, 2C) from mean-centered values. Stress increased at all daily TLs from 0.5 (11%) to 1.6 units (36%) with a biphasic response noted at 576 A.U (1.0–1.5 and 5.0 days, 2E) and 794 A.U (1.0–2.0 and 5.0 days, 2F). Soreness did not deviate significantly at 358 A.U (2G) versus mean-centered values, whereas fatigue (2J) showed some change (±0.5 units; ±10%) at this load. Both subscales responded similarly at the two highest loads. At 576 A.U, we saw a significant increase in soreness (0.6 to 1.7 units; 12 to 34%, 2H) and fatigue (0.8 to 1.6 units; 17 to 33%, 2K) after 0.5–2.0 days, with 794 A.U promoting a more dramatic rise in soreness (up to 3.7 units; 76%) and fatigue (up to 3.5 units; 72%) on lag periods from 0.5–3.0 days (2I and 2L respectively). A small biphasic rise in fatigue also emerged at 358 A.U (0.6 units; 12%), 576 A.U (0.9 units, 19%) and 794 A.U (1.1 units; 22%), all at a time lag of 5.0 days. Changes in sleep quality mirrored that of mood; declining significantly (-0.8 to -1.3 units; -13 to -21%) from mean-centered scores at a daily TL of 358 A.U (0.5–2.0 days, 2M), 576 A.U (1.0–2.0 and 5.0 days, 2N), and 794 A.U (5.0 days, 2O).

## DISCUSSION

This study explored daily TL and well-being associations across an international rugby series, including detailed characterization of delayed well-being effects at three different TLs. Descriptive profiling revealed substantial fluctuations in daily TL within and between study weeks, whereas the well-being subscales oscillated around a stable weekly baseline. The DLNMs identified delayed, but highly nuanced, effects of daily TL exposure on each well-being subscale. The well-being responses were distinguishable by differences in lag interval, duration, and magnitude of change, at each TL dosage.

Daily TL was highly variable across the international rugby series, in line with other tournament play or competition data [7, 13, 14, 15]. The large weekly variation in daily TL reflects, in part, a team-management strategy. Following the defeat in week one, a coaching decision was made to reduce training volume, leading to a -38% and -45% drop in daily TL over subsequent weeks. The quality of each match opponent is another consideration. Weeks one and two yielded similar match TLs against the 1^st^ and 2^nd^ ranked teams in the world (participant team was ranked 7^th^ and 8^th^), but a -30% drop in match TL arose in the last week against the 11^th^ ranked team. The daily TL shifts within a weekly macrocycle (i.e., +27% to -58% vs. Monday training) are typical of tapering strategies used in rugby to ensure peak performance on game day [15]. Whilst the day-to-day variation in TL was considerable across the rugby series, the well-being subscales exhibited smaller weekly fluctuations, before returning to a stable baseline. This consistency probably reflects the psychological processing of information, where internal drivers operate within tighter boundaries than external workload or TL measurements [3, 4, 7], coupled with narrower scales of measurement (e.g., 1–10 Likert) and stronger stability over time [13], as we demonstrated herein.

Cursory inspection of the DLNMs confirmed that physical stress can adversely affect mood state and quality of sleep, whilst promoting greater stress, muscle soreness, and general fatigue [2, 3, 4, 5, 12, 14]. More intricate patterns transpired at different TL dosages. For mood and sleep quality, a lag delay emerged at increasing daily TLs that speculatively reflects greater use of recovery strategies (e.g., naps, massage, contrast showers) following harder training days and matches. A rising daily TL also promoted a larger stress, soreness, and fatigue response, both in magnitude and duration. Soreness and fatigue were most reactive in this work, likely due to a combination of physical demands, bodily contacts and collisions [11]. The thresholding of the soreness subscale (i.e., no change at 358 A.U) can potentially be explained by stress habituation across the training camp, so that a stronger stimulus is needed to induce a perceptible change. The biphasic stress and fatigue response are also novel, but not widely reported in rugby literature [3] owing to study limitations (e.g., next-day comparisons). One possible reason is a cumulative training effect [3] that is intrinsic to our dataset. These nuanced patterns add to our understanding of well-being, as a multifaceted and dynamic process that adapts, transiently, to rugby stressors in intricate ways [1, 10, 11]. Also noteworthy is that the well-being changes are plausible (± 3.7 units) and predicted by TLs typical of an elite rugby environment, suggesting real-world interpretations and applications.

Erudition of the time-course of well-being recovery, with an incremental rise in daily TL, provides a stronger basis to guide decisions on team planning and management. For instance, a weekly TL can be better distributed to ensure that soreness and fatigue scores fall within an acceptable match-day range to optimize performance. This can be achieved by prescribing the heaviest training +3 days earlier, with implications for AM and PM load distribution based on the 0.5-day lag intervals that, if needed, can be condensed further (e.g., hourly) for more refined exercise prescription. The targeted recovery of soreness and fatigue might also prove expedient when a daily TL exceeds a nominal threshold (e.g., 1–2SD above the mean) for a given athlete and their anticipated recovery. Further possibilities exist to inform psychological-based strategies. As an example, a pre-match [26] or post-match [27] psychological intervention (e.g., player video footage, coach feedback) could help counter mood-related disturbances that manifest across a training week. Our findings also highlight the utility of the distributed lag (linear or non-linear) model, as a flexible approach to aggregate and explore complex bivariate associations in longitudinal sports data [28, 29, 30]. This includes simple, yet informative, plots to better communicate results to target audiences and data-driven estimates with real-world implications.

Several drawbacks of the current study are recognized. The selective recruitment of elite rugby players may limit knowledge transference to lesser-trained cohorts and to non-elite settings. Moreover, the TL and well-being data were collected on different time scales across the day, but for simplicity were modeled as time-matching variables. The next-day collection of match sRPE is another limitation. We also assumed that our well-being predictions are constant at the start and end of the series, and equivalent across all rugby activities. We do feel, however, that reasonable estimates were obtained once aggregating data across the rugby series. Further bias might arise from positional (i.e., forwards, backs) differences in rugby match demands and weekly workloads [1, 13, 14]. Sensitivity analyses (data not shown) indicated that the addition of positional group did not improve our models. Sensor-derived measures (e.g., global positioning, accelerometry) of player load were not available during this study, but would advance future work. We envisage other benefits by study replication across longer rugby tournaments and different seasonal phases, such as the partitioning of stress loads (and ensuing recovery) into periods of training only and training plus matches, as well as co-validation and refinement of our predictions for broader use in sport.

## CONCLUSIONS

This study offers new insight regarding physical stress and temporal dependencies in athlete well-being during an international rugby series. Daily TL exposure predicted adverse responses (i.e., declining mood and sleep quality, rising stress, soreness, and fatigue) that differed in time lag, lag duration, and magnitude, relative to dosage. A more precise understanding of these associations can guide training prescription and psychological strategies to optimize recovery, performance, and team success. Examples include better distribution of a weekly TL and the targeted recovery of soreness and fatigue, above an individual TL threshold.

## Data Availability

The data collected is not publicly available due to ethical restrictions.
